# Recent developments in speciation and determination of arsenic in marine organisms using different analytical techniques. A review

**DOI:** 10.1039/d4ra03000a

**Published:** 2024-07-08

**Authors:** Bashdar Abuzed Sadee, Yaseen Galali, Salih M. S. Zebari

**Affiliations:** a Department of Food Technology, College of Agricultural Engineering Sciences, Salahaddin University-Erbil Erbil Kurdistan Region Iraq bashdar.sadee@su.edu.krd; b Department of Animal Resource, College of Agricultural Engineering Sciences, Salahaddin University-Erbil Erbil Kurdistan Region Iraq; c Department of Nutrition and Dietetics, Cihan University-Erbil Erbil Iraq

## Abstract

Marine organisms play a vital role as the main providers of essential and functional food. Yet they also constitute the primary pathway through which humans are exposed to total arsenic (As) in their diets. Since it is well known that the toxicity of this metalloid ultimately depends on its chemical forms, speciation in As is an important issue. Most relevant articles about arsenic speciation have been investigated. This extended not only from general knowledge about As but also the toxicity and health related issues resulting from exposure to these As species from the food ecosystem. There can be enormous side effects originating from exposure to As species that must be measured quantitatively. Therefore, various convenient approaches have been developed to identify different species of As in marine samples. Different extraction strategies have been utilized based on the As species of interest including water, methanol and mixtures of both, and many other extraction agents have been explained in this article. Furthermore, details of hyphenated techniques which are available for detecting these As species have been documented, especially the most versatile and applied technique including inductively coupled plasma mass spectrometry.

## Abstract 2

Various available techniques and separation methods are vital for measurement of arsenic species in marine samples.

## Graphical abstract

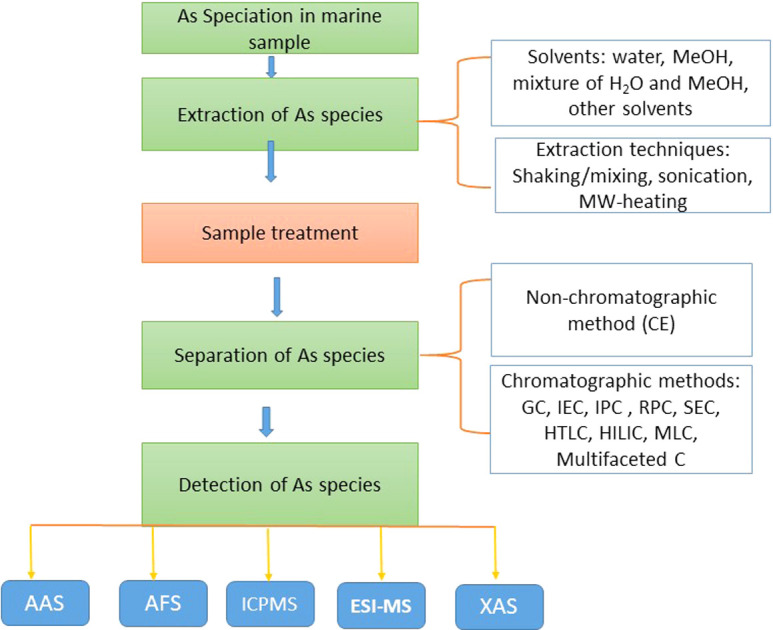

## 1 Introduction

Seafood constitutes a diverse group of aquatic organisms, including those from both marine and freshwater environments such as mollusks, crustaceans, and various types of finfish. While there is consistent evidence supporting the health benefits of moderate seafood consumption, concerns arise due to potential risks and negative impacts associated with inherent contaminants, particularly organic arsenic (As) species, with As being a notable example. This has prompted apprehension about the consumption of aquatic foods. Seafood and seaweed serve as primary dietary sources of total arsenic in humans, predominantly in the form of organic arsenic (oAs) species. However, exceptions exist, with elevated levels of inorganic As (iAs) reported in specific instances, such as in the edible seaweed Hijiki (*Hizikia fusiformis*), freshwater fish from Thailand, and blue mussels from Norway. Among seafood exposure sources, marine algae and shellfish exhibit the greatest diversity of arsenicals.^1,2^

When heavy metals are accumulated and taken up by plants in edible and non-edible fractions at a particular level, both animals and people may experience health issues.^2^ Environmental trace elements are harmful to human health. Numerous studies have demonstrated that these factors can have an impact on the environment and the quality of food. It is commonly recognized that heavy metal contamination can pollute human food supplies, particularly vegetables.^3^ Determining the quantity of certain metallic elements is crucial since consuming large amounts of these elements is harmful.^4^

As, with an atomic number of 33, is widely acknowledged for its harmful effects on both human beings and marine animals.^5^ As, ranked as the 12th most abundant metalloid in the human body finds widespread applications in various industries such as agriculture, electronics, metallurgy, and the production of chemical weapons, cattle, insecticides, fertilizers, and medicinal compounds.^6,7^ When As, present in the earth's crust at 1.8 ppm by weight, combines with oxygen, chlorine, and sulfur, it forms iAs compounds. Rock–water interactions are the primary contributors to the release of As and the degradation of groundwater quality in aquifer systems. As commonly exists in three allotropic forms: black, yellow, and grey.^6^ The concentrations of As in unpolluted fresh and sea water are < 1 to 10 μg L^−1^ and from 1 to 3 μg L^−1^, respectively.^8^

The primary source of environmental As contamination stems from human activities, posing a severe threat to millions of people who face life-threatening complications due to the consumption of water tainted with As or the consumption of food grown in As-contaminated soils or irrigated with As-laden water. Researchers and authorities have identified As contamination as a catastrophic issue spanning the twentieth to twenty-first century.^9,10^ As pollution acknowledged as a human carcinogen, impacts hundreds of millions of individuals globally. iAs stands out as a leading factor in the development of skin, lung, bladder, liver, prostate, and kidney cancer in humans.^11^ The biogeochemical dynamics of As are controlled by various physical–chemical processes, such as oxidation–reduction, precipitation/solubilization, and adsorption/desorption, along with biological mechanisms, including microbiological processes.^12^

Thus, this review provides an overview of the techniques employed for extracting As species in marins samples. Subsequently, these species are separated using both chromatographic and non-chromatographic tools, followed by the determination of As levels and the identification of its various forms using hyphenated techniques.

## 2 As species

The toxicity, bioaccumulation, and mobility of As are significantly influenced by both the chemical form and the degree of methylation.^13,14^ There are numerous inorganic and organic forms of As with different toxicity characteristics. As^0^ (metalloid arsenic, 0 oxidation state), As^III^ (trivalent state, *e.g.* arsenites), As^−III^ (trivalent state, arsine and arsenide, −3 oxidation state) and As^V^ (pentavalent state, *e.g.* arsenates) are the three common valence states in which it can be found.^7,15^

### 2.1 Inorganic arsenic

The existence of iAs (including both As^III^ and As^V^) is recognized as a factor contributing to As exposure through the consumption of seafood. In the majority of seafood, concentrations of iAs are minimal. Nevertheless, the brown algae known as hijiki (*Sargassum fusiforme*), an edible seaweed commonly utilized in Asian cuisine, is widely acknowledged for containing elevated levels of total As, with the predominant form being inorganic.^16,17^ The distribution of iAs in seaweed is influenced by taxa, with some species exhibiting higher proportions. In specific locations, increased levels of iAs have been observed in bivalves and gastropods, prompting the establishment of consumption guidelines in the Pacific US.^18^ Increased levels of iAs in the bodies of organisms may align with higher concentrations of As in sediments and the water column. This correlation is contingent on the feeding mechanism of the organisms and can be attributed to their proximity to a point source of contamination.^18,19^ In contrast, the concentrations of oAs in seafood remain unaffected by the presence of contamination at sites.^18^ Pelagic fish, characterized by a typically low proportion of iAs, do not tend to accumulate higher concentrations of total arsenic in regions with elevated As levels. On the other hand, benthic-feeding organisms have the potential to accumulate increased concentrations of iAs.^19,20^ While efforts to evaluate exposure to iAs from seafood have increased compared to oAs, there still exists considerable uncertainty in predicting levels of iAs in various marine-derived food items.^21^

### 2.2 Methylated As compounds

Methylated arsenic compounds are found in marine ecosystems due to the enzymatic methylation of iAs, resulting in compounds with 1–4 methyl groups. These compounds are usually present as minor arsenic species in seafood, with dimethylarsinic acid (DMA) being the most prevalent. Mollusks may contain DMA in higher proportions compared to what is typically observed in finfish or algae.^22–24^ Monomethyl arsenic acid (MMA) is only present at trace levels and is not common in marine ecosystem.^25^ The trimethylated form, trimethylarsine oxide (TMAO), is another minor compound that has not been identified in significant amounts in seaweeds. However, it has been isolated in a variety of aquatic creature species and freshwater fish, usually found in trace levels^25–27^ TETRA is a less prevalent As compound in finfish and freshwater fish but serves as the primary species in various mollusks. Reported amounts range from 0.2 to 16 μg g^−1^ in different organs of certain clams.^25,26^

#### 2.2.1 MMA^III^ and DMA^III^

Low quantity of methylated arsenicals can be found in marine foods. Methylarsenicals are synthesized in marine environments by bacteria, phytoplankton, and microbial degradation of plant material from iAs. They are then transferred into to seafood and other food chain.^28^ Nevertheless the metabolic pathways of methylation of iAs in humans has not been clearly explained, it is considered as a detoxification for a period of time.^29^ Because MMA^III^ and DMA^III^, which are more toxic than iAs, are produced from biotransformation of iAs should be considered as a detoxification process in microorganisms.^30,31^ MMA^III^, which has long been considered of as a transient intermediary in the methylation pathway, is really a stable metabolite of iAs and has been observed in hamster liver, rat bile, and human urine in significant (*e.g.*, effect levels) amounts after exposure to iAs.^32,33^

The nutritional status of phytoplankton and its boost phase are linked to the elongation of methylarsenicals. During the lag period of phytoplankton growth, production of DMA^V^ steadily increases whereas DMA^III^ and MMA^III^ remain relatively stable.^34^ It has been proposed that MMA^III^ hazardous and toxic to liver, skin and lung cells than As^III^. Furthermore, DMA^III^ is more hazardous than DMA^V^ and As^V^, therefore, it can enter cells very rarely because of having negative charge.^35,36^ As^III^ and As^V^ are more cytotoxic than methylated pentavalent arsenicals MMA^V^ and DMA^V^, while methylated trivalent arsenicals namely MMA^III^ and DMA^III^ are more cytotoxic than corresponding As^III^ and As^V^.^37^

### 2.3 Arsenobetaine

In marine organisms, iAs has the potential to undergo bioconversion into methylated species such as MMA or arsenobetaine (AsB). AsB typically emerges as the predominant end product in As metabolism within marine organisms. The rate of biotransformation is contingent on the As uptake and transformation mechanisms specific to the animals. Conversely, studies on freshwater fish present conflicting findings. Some literature indicates lower concentrations of total arsenic (TAs) in freshwater fish samples, while others report elevated TAs levels with substantial proportions of iAs. Certain authors argue that AsB is a predominant species in freshwater fish samples.^25^ AsB predominates as the major As species in most finfish and shellfish. It is also present in zooplankton and prevalent in certain algae forming the foundation of the food web, but its occurrence in algae may potentially be attributed to epiphytic plankton or bacteria on the surface of marine flora.^38,39^

In bivalve mollusks, where As speciation is intricate, AsB can constitute a substantial portion of water-soluble As. In contrast, in cephalopods and crustaceans, characterized by simpler As speciation, AsB emerges as the predominant species.^22,23,40,41^ In finfish, AsB is also the primary As species, although arsenolipids (AsLipids) can constitute a significant fraction in certain oily fish.^42,43^

### 2.4 Arsenosugar

**Fig. 1:**
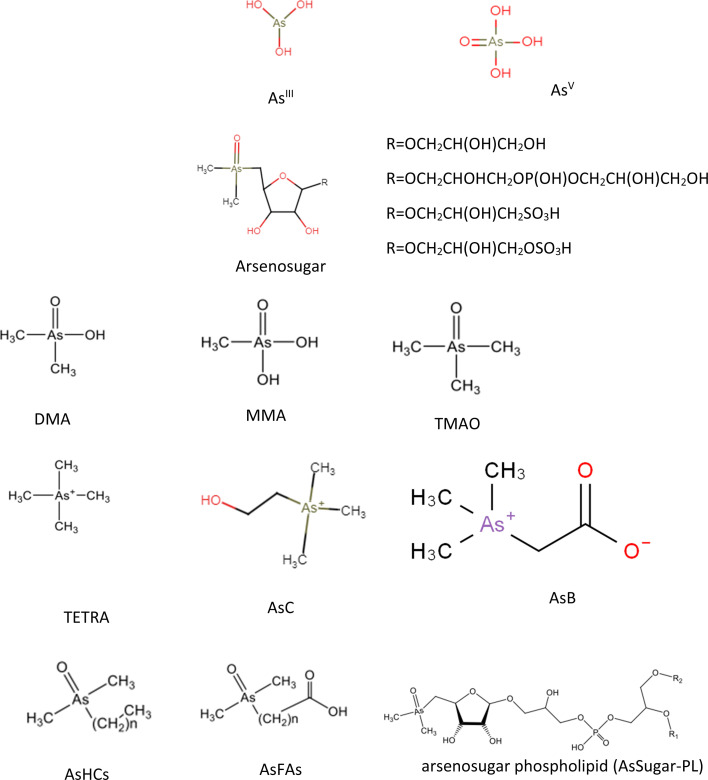
The chemical structure of the most common As species in marine samples.

The predominant species in most genera of algae are ribofuranosides containing As, commonly referred to as arsenosugars (As-sugars). As-sugars play a crucial role in the transformation and cycling of As in the marine environment, and these mechanisms have been subject to study while As-sugars may not exhibit acute toxicity, there is a potential for mild chronic toxicity. Given the high consumption of seaweed, evaluating exposure to various As-sugars is essential. However, as of now, there is limited reliable information available on their toxicity.^44^ To date, there are at least 20 identified As-sugars compounds, with four of them being significantly prevalent in a wide range of marine organisms (Fig. 1). As yet, no biological function for As-sugars has been clearly defined, and their exact biosynthesis remains unknown. The enzymes responsible for attaching the ribose moiety to DMA (dimethylarsinic acid) have not been identified, although there is a strong likelihood that the methyl groups and ribose moiety attached to arsenic are supplied by S-adenosylmethionine (SAM).^45^ Phytoplankton and the brown macroalgae *Fucus serratus* are responsible for direct synthesize of As-sugars.^46,47^ As sugars have been detected in mussels from deep-sea vents, indicating a potential bacterial origin for these compounds and freshwater fishes.^25,48^

### 2.5 Arsenocholine

Arsenocholine (AsC) serves as a metabolic precursor for AsB in aquatic animals. AsB is formed from the inclusion of labeled AsC when it is taken up with lesser quantity of iAs, MMA, and/or DMA.^49^ It is believed that the degradation of As-sugars leads to produce the non-toxic AsC which is mainly found in aquatic animals. The conversion of AsC to AsB is achieved by the GbsB and GbsA, the enzymes encoded by the gbsAB glycine betaine synthetic operon in the rhizobacterium *Bacillus subtilis*. This process takes place *via* successive oxidation reactions: where first GbsB oxidizes AsC to AsB aldehyde, which is then more oxidized to AsB by GbsA. On the other hand, the pathway of the production of AsC from As-sugars still remains unclear.^50^ According to the results of As species obtained in the laboratory degradations and feeding studies, AsC has been proposed to form *via* the decomposition of dimethylarsenoribosides, where a range of dimethylarsinoyl-hydroxycarboxylic acids and the corresponding thio-arsenic components are produced as intermediates. This is based on As species found in laboratory degradation and feeding studies.^51^ Freshwater fish could serve as a significant source of various As compounds, including AsC.^25^

### 2.6 Arsenolipids (AsLipids)

Seafood, including fatty fish,^52^ freshwater fish,^25^ algae,^53^ and crustaceans,^54^ exposes humans to arsenolipids, or AsLipids. On the other hand, little is known about these chemicals' toxicity, identification, and abundance.^52^ AsLipids, which comprise fatty acids (AsFA), hydrocarbons (AsHC), and glycophospholipids (AsPL), are another class of As chemicals found in seafood. Phosphatidylcholines, phosphatidylethanolamines, and alcohols containing As have also been discovered, however AsLipid compound characterisation is still far from enough. The distribution of AsLipids in seafood is mostly unknown, although these substances are typically connected to oily fish and fish oils. AsLipids, primarily AsPL and AsHC, have been reported to make up 1.6–6.7% of As in brown algae. However, the exact percentages may differ amongst taxa.^55,56^ A study showed that the level of AsLipids in demersal fish was lower than pelagic^43^ and high levels, ranging from 50 to 62%, have been observed in the fillet of oily fish.^42,43^ The quantity of lipophilic As present may affect the relative amounts of AsHC and AsFA in fish, with AsHC being present in fish with higher AsLipid concentrations.^57^ AsLipids make up as much as 70% of the total arsenic concentration in seafood.^43^ The highest concentrations can be found in fatty fish, such as mackerels and herring. Arsenolipids (AsLipids) are thought to ascend the food chain, beginning from algae to higher organisms like fish, with the potential for endogenous synthesis within the organism given the similarities between the identified As-containing fatty acids (AsFAs) and common fatty acids present in aquatic organisms.^52^ Diverse aquatic systems, including herring,^43^ tuna,^42^ cod,^58^ fish oils,^59^ and edible brown algae ^55^ contain As-containing hydrocarbons (AsHCs).

## 3 Toxicity of As species and health hazards

The WHO deemed this As poisoning to be the “largest mass poisoning of a population in history” because Bangladesh as a whole experienced the worst As poisoning public health danger.^60^ The global presence of As in its natural or geogenic form poses a widespread issue with a diverse range of health effects on both humans and wildlife. Because iAs is hazardous and does not actually reflect any helpful metabolic functions, it can cause diseases of the skin, circulatory system, neurological system, and even cancer.^61^ Furthermore, the water contaminated with As leads to the existence of iAs in the diet. The consumption of food represents a significant route of exposure, and extended exposure to water containing high concentrations (>100 mg L^−1^) of iAs associates with non-melanoma skin, lung, and bladder cancers.^62^

In recent times, elemental speciation has become a widely recognized area of research. An element's toxicity, mobility, and biological availability can all be better understood by looking at it in its chemical form.^63^ The iAs species, encompassing As^III^ and As^V^, are classified as cancer-causing agents.^64,65^ On the other hand, organic As species, including MMA and DMA, are considered less toxic than iAs but are still categorized as cancer-provoking agents. In contrast, AsC and AsB are classified as non-toxic As species.^66^

As is among the numerous carcinogens that induce severe diseases impacting the integrity of human cells and genetic materials upon exposure. By interacting with protein sulfhydryl groups and substituting arsenate for phosphate groups, inorganic arsenicals cause toxicity.^67^ Numerous health problems, such as cancers, cardiovascular effects, pulmonary, immunological, and endocrine disorders, reproductive health effects, neurological disorders, liver disease, gastrointestinal disturbances, genotoxicity, arsenicosis, and dermal infections have been related to these inorganic arsenical poisonings.^68,69^ As is commonly believed to undergo methylation predominantly as a means of reducing its toxicity, viewed as a detoxification process. However, as of 2020, it was revealed that the formation of methylated metabolites containing trivalent As was essential for causing certain adverse effects associated with As exposure. These findings align with alterations in arsenic's dynamic behaviour resulting from the methylation process. Because the trivalent oxidation state of As is correlated with heightened efficacy as a cytotoxin, clastogen and may create a hazardous pathway that encourages detrimental biological processes, leading to gastrointestinal problems, cancer, and skin diseases.^70–72^ In line with empirical findings, scientific research supports the notion that As negatively impacts neurodevelopment and causes birth problems, even at low concentrations when exposure occurs during early life.^62^ Exposure to As contamination during pregnancy has been identified as a factor contributing to changes in gene expression pathways linked to diabetes. This association increases the risk of developing diabetes in adulthood.^73^ Exposure to As can have detrimental health effects on both humans and other living organisms. The potential side effects encompass a range of issues, including alterations in skin conditions, respiratory problems, cardiovascular issues, disturbances in the digestive system, as well as genotoxic, mutagenic, and carcinogenic effects.^74^ In cases of acute toxicity, As, a toxic metalloid, can lead to symptoms such as nausea, vomiting, and severe diarrhea. In contrast, chronic toxicity is associated with more severe health consequences, including cardiovascular disease, diabetes, bladder cancer, and kidney cancer.^75^ The adverse health effects of As exposure are diverse and include various malignancies (lung, bladder, kidney, skin, and liver), neurological disorders, cardiovascular diseases, hypertension, gangrene, diabetes, respiratory diseases, renal diseases, and reproductive issues.^76^ The main variables affecting the severity of As poisoning are the quantity of As consumed, nutritional state, duration of exposure, and immune response of the individual. The obvious symptoms of long-term As exposure include skin lesions like arsenicosis. Furthermore, arsenicosis is a not the issue of individual countries but it is also a global one.^77^

## 4 Risk assessment of As exposure from marine samples

Recommendations have been made by numerous international organizations regarding the maximum quantity of As that food should contain. This is because As has a negative impact on human health due to its significant enrichment and biotransformation. As is toxic to humans and can have an adverse effect on people of any age or health state. The class of As with the highest potential for toxicity is iAs. As the Food and Drug Administration (FDA) keeps surveillance and regulates amounts in food, dietary supplements, and cosmetics. As can only be reduced in food, but it cannot be totally eliminated or prevented. As in food items does not yet have a maximum limit set by the European Union (EU).^72^ The Scientific Panel on Contaminants in the Food Chain (CONTAM Panel) of the European Food Safety Authority (EFSA) issued an opinion on As in food on October 12, 2009. In this opinion, the CONTAM Panel asserted that the provisional tolerable weekly intake (PTWI) of 15 μg kg^−1^ body weight, as established by the Joint FAO/WHO Expert Committee on Food Additives (JECFA), is no longer suitable. This reassessment was based on data indicating that iAs is linked to lung and urinary bladder cancers, as well as skin cancer. Additionally, adverse effects have been observed at exposures lower than those considered by JECFA. For skin lesions, bladder cancer, lung cancer, and skin cancers, the CONTAM Panel determined a range of benchmark dose lower confidence limits (BMDL01) values ranging between 0.3 and 8 μg kg^−1^ body weight per day. EFSA proposed maximum values for iAs in various types of rice, including non-parboiled milled rice (polished or white rice), parboiled rice and husked rice, rice waffles, rice wafers, rice crackers and rice cakes, and rice for the manufacturing of foods for infants and children. The proposed maximum values were set at 200, 250, 300, and 100 μg kg^−1^, respectively.^78^

As is identified as one of the contaminants of emerging concern in seafood. However, there is insufficient data available on the levels of As in seafood to conduct a comprehensive risk assessment.^79^ In order to address harmful As in food and to produce more speciated As data, EFSA produced a risk profile for arsenicals in diet.^80^ Currently, risk associated with As from seafood can only be assessed based on the iAs species. Some seaweeds, where taxa largely determine As concentration, and some bivalves, where iAs concentration is linked with the location of harvest, have been identified as vital exposure risks for iAs. For organic As, a task of exposure risk is currently not possible – there are simply not sufficient data on levels and compartments of various species in seafoods, and there is an almost complete lack of toxicity data and human population studies. The data available, however, demonstrate high concentrations of organic As, present as a wide range of species, in seafood, and indicate metabolism and toxicity of some of these oAs compounds.^81^

Since As is involved, As^III^ is a well-established carcinogen. As-containing hydrocarbons such as AsHC 332, AsHC 360, and AsHC 444, As-containing fatty acids such as AsFA 362 and AsFA 388 (Fig. 1), and methylated trivalent arsenicals such as MMA^III^ and DMA^III^ are among the other dangerous arsenicals. Additional toxicity research is required for the recently discovered organoarsenicals to identify all potential sources of hazards. These studies aim to determine the severity and frequency of associated adverse health effects. It is important not to restrict toxicity investigations solely to the organoarsenicals but to extend them to their metabolites. This is crucial because it has been established that the acute toxicity of most arsenicals is limited, and toxicity often arises from metabolic transformations. For instance, As-sugars have metabolites resembling As^III^, a recognized carcinogen. Numerous organoarsenicals remain untested for toxicity and assumptions of their non-toxic nature are based on the benign characteristics of some well-known organoarsenicals like AsB. Nevertheless, the toxicity status of these compounds is not established. Therefore, it is crucial to conduct toxicity studies for these recently identified organoarsenicals.^81^

Many countries are currently assessing or contemplating food restrictions based on As evaluations. Given its well-documented toxicity, relevant epidemiological evidence from studies on drinking water, and ample datasets demonstrating its presence in various foods, iAs has understandably been the primary focus in these assessments. However, for a comprehensive evaluation of As in food, it is crucial to consider oAs compounds present in seafood.

## 5 Effects of freezing, cooking and processing on arsenic species

Foods are inevitable subjected to many processing including cooking, freezing drying and many more. It is crucial to understand their effects on As fate in food after the processing. A study was conducted to evaluate the stability of As compounds in seafood samples during processing and storage by freezing. It was concluded that the AsB and total As decreased in blue musseles as a result of freezing and storage, meanwhile the content of these As species was not changed in Atlantic cod and Atlantic salmon.^82^ The amount of As that is actually consumed might vary significantly depending on how food is processed. For example, the custom of washing and soaking the seaweed *Hizikia fusiforme*, which is well-known for having high iAs content, can reduce it by up to 60%,^83^ meanwhile the concentration of total As or iAs in certain types of seafood may increase due to the loss of water during the cooking process.^84^ Heating including frying probably leads to transformvof AsB to TETRA as a result of decarboxylation of AsB. The concentration of DMA in boiled and fried finfish was significantly higher than that of raw samples, they thought of this as a result of decomposition of other As species.^82^ However, in another study the content of DMA decreased as a result of boiling of marine animals including fish, shellfishes, shrimp, sea anemones and squid9. They assumed that DMA is converted to As^V^ during cooking.^85^

## 6 As speciation in marine food origin

**Fig. 2:**
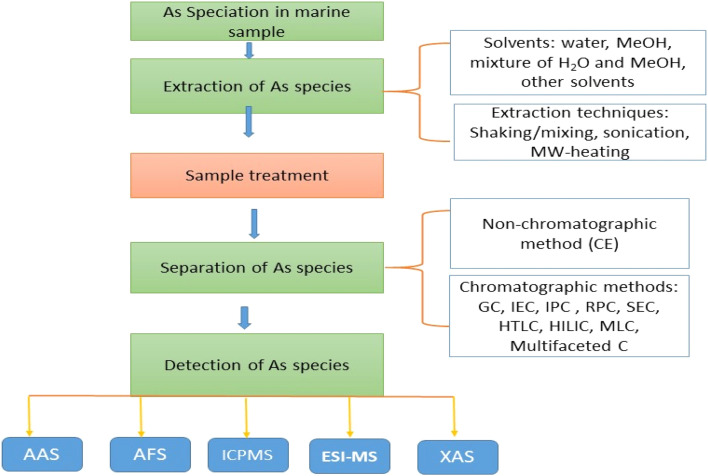
Summary of As speciation in marine samples.

Ideally, to obtain precise information on As speciation, it is crucial to preserve the concentration and chemical composition of the original species throughout the sample preparation, extraction procedures and also compatible with the contracted separation and detection methods^7,86^ (Fig. 2). The choice of an extraction method for a specific application is affected by both the matrix and the target species. To accurately evaluate As species using HPLC-ICP-MS, it is crucial to employ a gentle and efficient extraction procedure.^87^ There isn't a universal extraction method convenient for all samples and As species, as most researchers are already attentive. This doesn't decline the importance of the field of sample extraction; rather, it emphasizes that the sample extraction procedure should be adapted based on the specific application and objectives of the study. These considerations will change from one study to another, depending on a range of factors. Eventually, finding the most effective extraction procedure for a particular group of samples is an essential component of a comprehensive study aimed at providing information about As species.^88^ Because of the complexity of As speciation, a more practical approach for high-throughput monitoring may involve grouping As species into fractions based on their toxicity, such as AsB, As-sugars, and iAs.^89^

### 6.1 Solvents

The speciation of As-sugars may not be vital; instead, there is a demanding need for robust methods that target As forms, including iAs and AsB, along with a fully quantifiable non-speciated As fraction. This approach is important for collecting extensive datasets that can lead legislative attempts, authorize guideline standards, and dispatch the issue of toxic As in marine-origin.^89^

Desirable high extraction efficiencies are contingent not only on the forms and type of tissue under study but can also show variability among various species within the same family.^90^ As an illustration, fish tissues exhibited extraction rates of 90–100%,^91^ whereas oyster, red algae, and brown algae demonstrated extraction efficiencies ranging from 85–100% when using a water/methanol mixture.^92^

Typical extraction solvents utilized for marine samples comprise ultrapure water, methanol–water mixtures, hexane, dilute acids, and chloroform. Several methods, employing a combination of both polar and nonpolar organic solvents as extractants, have been documented to successfully extract As species from seafood. For instance, a combination of methanol/dichloromethane (DCM) and methanol/chloroform mixtures has been separately utilized to extract AsLipids from fish. Ultrapure water is considered the most suitable extractant for speciation analysis due to the polar nature of most As species. However, as a soft extractant, water may not be able to extract all As species, particularly because of the presence of lipophilic arsenicals in seafood.^90^ Methanol is widely used as an extractant for seafood due to its limited co-extraction of non-arsenicals and its ease of removal through evaporation.^88^ Methanol (MeOH), H_2_O, or combination of both utilize frequently to extract the water soluble As species.^93^ The methanol–water mixture affords a convenient balance between the solubility of As and the ease of solvent removal. This is because the majority of naturally occurring arsenicals in seafood are polar and water-soluble.^93^ Nevertheless, the recovery of As compounds through this method may be limited, especially for marine algae and oily or fatty fish containing high proportions of nonpolar arsenicals.^94,95^

Extractions performed under acidic conditions are documented to obtain higher extraction efficiencies, likely due to acid hydrolysis causing the releasing of degradation products from As species within lipid and protein fractions.^87,96^ This harsh acidic circumstances can also induce the degradation of different As-sugars into a singular riboside compounds.^96^ Tetramethylammonium hydroxide (TMAH) has been utilized for the extraction of As-sugars, a challenging burden in oysters and shellfish, resulting in improved extraction efficiencies. Similar riboside cleavage to provide trace amounts of DMA has been seen in basic extractions along with similar degradation of As-sugars.^97^

Since enzymes may selectively release analytes from the sample matrix without causing species transformation, they have been used in speciation analysis. This is because enzymes can break down specific bonds in the substrate at mild pH and temperature settings.^22,98^ For instance, enzymes like trypsin, pancreatin, and phospholipase D have been employed in As speciation extraction.^99–101^ Trypsin, being a proteolytic enzyme, has found application in As speciation studies involving various fish species, including (ling, gurnard, grey mullet, pollock, dover sole, john dory, megrim, flounder, dab, sand sole, brill, lemon sole and halibut.^87^ Enzymes can be employed to simulate living conditions, such as the digestive processes in the stomach, in order to ascertain the bioavailable portion of a species.^102^ It has been shown that artificial gastric juice is more effective at extracting As species than commonly used extractants such methanol–water, ultrapure water, and dilute HNO_3_ (0.15 M).^103^ The lengthy incubation period—typically 12 to 24 hours—the requirement to incubate in a bath at 37 °C, and the comparatively high cost of the reagents are general drawbacks of conventional enzymatic hydrolysis that restrict its applicability in speciation studies.^104^ Nonetheless, the extraction time is greatly shortened when microwave-assisted extraction (MAE), pressurized liquid extraction (PLE), or ultrasound probe sonication (UPS) are combined with enzymatic hydrolysis.^105–107^

### 6.2 Extraction system

Different approaches and techniques are used in extracting As species from marine dietary sources. Environmental considerations, including the low toxicity of the extractants and minimal waste production, have been instrumental in advancing classical extraction techniques. This lead to achieve faster, more reliable, and environmentally friendly extraction methods.^108^

The conventional method for sample extraction involves solvent extraction, employing various solvents and/or solvent mixtures, using techniques such shaking, heating, magnetic stirring, sonication, and microwave have been used.^109^ Due to its long extraction periods, high solvent volume usage, and low preconcentration factors, solvent extraction is less commonly employed.^102^ Nevertheless, advanced extraction techniques have been employed to minimize solvent usage. Examples of these techniques include supercritical fluid extraction (SFE), accelerated solvent extraction (ASE), PLE, and MAE.^110,111^ Additionally, solvent-free methods such as solid-phase microextraction (SPME) or sorbent extraction phases, as seen in matrix solid-phase dispersion (MSPD), along with physical treatments like UPS, are employed. Because MAE offers acceptable and consistent efficiency, shorter extraction times, less solvent use, and the capacity to do many extractions rapidly, it is a useful alternative to traditional methods for many matrices. This approach has been utilized in many studies on the speciation of As. Optimization is straightforward because there aren't many factors to consider, including matrix properties, solvent volume, temperature, extraction time, power, and solvent selection.^112,113^ When it comes to extracting organic analytes from complex matrices, such as seafood, the majority of advanced extraction techniques have proven to be more efficient.^108^

Table 1 provides a summary of the main extraction media and extraction techniques utilized to remove As species from marine samples. It should be noted that several of these procedures have fairly low extraction efficiency and are typically time-consuming.

**Table 1:** Summery of the most commonly used extraction procedures for extraction of As in marine samples^a^

Matrix	Extraction media	Extraction technique	Technique	Reference
Marine macroalgae and herbivorous animals	H_2_O	Deionized water was used to dissolve residue and employing HNO_3_ and H_2_O_2_ in a water bath at 90 °C	HPLC-ICP-MS	114
Kelp powder and marine bivalve	H_2_O	Shaking	HPLC-ES-MS/MS	115
Fish (silver bream, bream, trout, sturgeon) and crab	H_2_O	Water was used to extract As species using MAE	HPLC/ICP-DRC-MS	25
Fish	H_2_O	Shaking/mixing	HPLC-ICP-MS, HPLC-ES-MS	115
Crustaceans	H_2_O	Shaking overnight	HPLC-ICP-MS	116
Algae	H_2_O	Shaking overnight	HPLC-HG-ICPMS	117
Algae	H_2_O	Shaking for 16 h	LC-ICP-MS	118 and 119
Cod, haddock, mackerel, crab, shrimp, geoduck clam, oyster, and kombu	Hot water	Deionized water, vortex, heating up to 90 °C over 45 min and hold for 30 min	LC-ICP-MS	120
Algae, crustaceans, molluscs and fish	H_2_O	Sonication	HPLC-ICP-MS	121
Crustaceans and molluscs	H_2_O	Shaking/mixing + sonication	LC-ICP-MS	48
Marine macroalgae and herbivorous animals	CH_3_OH/H_2_O	50% (V/V) methanol-deionized water using MAE and heated to 75 °C for 10 min	HPLC-ICP-MS	114
Clam, oyster, cuttlefish, shrimp and finfish	CH_3_OH/H_2_O	Methanol/water (1/1, v/v), shaking overnight	LC-ICP-MS	120
Fish	CH_3_OH/H_2_O, 1 : 9 (v/v)	Shaking	HPLC-ICP-MS	122
Algae	CH_3_OH/H_2_O, 1 : 9 (v/v)	Shaking	HPLC-ICP-MS	123
Fish, molluscs, algae	CH_3_OH/H_2_O, 1 : 4 (v/v)	Shaking/mixing	HPLC-ICP-MS	124
Crustaceans	CH_3_OH/H_2_O, 1 : 4 (v/v)	Shaking/mixing	HTLC-ICP-MS	125
Fish, molluscs and crustaceans	CH_3_OH/H_2_O, 1 : 1 (v/v)	Shaking	HPLC-ICP-MS	126
Carb, shrimps, benthic fish, and pelagic fish	CH_3_OH/H_2_O, 1 : 1 (v/v)	The extract was heated to 50 °C to evaporate the solvent until a volume of approximately 1 mL was reached	HPLC-UV-HG-AFS	127
Algae	CH_3_OH/H_2_O, 1 : 1 (v/v)	Shaking	HPLC-ICPMS	128
Algae, fish, molluscs	CH_3_OH/H_2_O, 1 : 1 (v/v)	MAE-heating	HPLC-ICPMS	128
Molluscs	CH_3_OH/H_2_O,^111^ 2 : 1 (v/v)	Shaking/ultrasonic	HPLC-ICP-MS	129
Algae	CH_3_OH/H_2_O, 80 : 20 (v/v)	Shaking	IC-ICP-MS	130
Fish, molluscs	CH_3_OH/H_2_O, 4 : 1 (v/v)	MAE-heating	CE-ICP-MS	131
Fish	CH_3_OH/H_2_O, 90 : 10 (v/v)	Shaking overnight	HPLC-HG-AFS	132
Molluscs	CH_3_OH/H_2_O, 10 : 1 (v/v)	Shaking at 30 °C	HPLC-HG-AFS	133
Algae, crustaceans and fish	CH_3_OH/H_2_O, 1 : 1 (v/v)	Sonication	HPLC-ICP-MS	134
Molluscs	CH_3_OH/H_2_O, 1 : 1 (v/v)	Sonication	HPLC-UV-HG-AFS	135
Mussels	CH_3_OH	Shaking/mixing at ambient temperature	HPLC-ICPMS	136
Algae, crustaceans and fish	CH_3_OH	MAE-heating	HPLC-GF-AAS	137
Fish	Trypsin in 0.1 mol L^−1^ NH_4_HCO_3_	Shaking in water bath at 37 °C for 12 h	HPLC-ICP-MS	87
Crustaceans, fish and molluscs	(1) Acetone	MAE-heating	HPLC-ICP-MS	138
	(2) CH_3_OH/H_2_O, 1 : 1 (v/v)			
Molluscs	(1) Acetone	A two-step sequential extraction with acetone and MeOH/water was used with the aid of shaking/mixing	HPLC-ICP-MS	130
	(2) CH_3_OH/H_2_O, 1 : 1			
Marine macroalgae and herbivorous animals	Acetone, HNO_3_	Agitated on a mixing wheel for 1 h, heating using a hot water bath (90 °C)	HPLC-ICP-MS	114
Crustacean	(1) Hexane	Hexane extraction was added to a mixture of methanol/Milli-Q water (9 : 1 (v/v); 15 mL) and shaken for 12 h	HPLC-ICP-MS	139
Fish	(2) CH_3_OH/H_2_O (9 : 1 v/v)			
Shark; shrimp; squid; oyster; scallop	10 mmol L^−1^ (NH_4_)_2_HPO_4_	Shaking	LC-ICP-MS/MS	140
Clam, oyster, cuttlefish, shrimp and finfish	Trifluoroacetic acid	0.1 M trifluoroacetic acid solution containing 1% (v/v) of a 30% (w/w) hydrogen peroxide solution were added (for inorganic As species)	HPLC-ICP-MS	126
Clam, oyster, cuttlefish, shrimp and finfish	DCM/CH_3_OH, acetone, formic acid, NH_3_, ethanol	DCM/CH_3_OH (2 : 1 (v/v)), DCM/acetone (1 : 1, (v/v)), DCM/acetone 1% formic acid, 3 mL of CH_3_OH 1% formic acid and CH_3_OH 1% aqueous NH_3_), ethanol	HPLC-ICP-MS	126
Cod, haddock, mackerel, crab, shrimp, geoduck clam, oyster, and kombu	Dichloromethane–CH_3_OH	Mixture and gently shaking at room temperature for 60 min	LC-ICP-MS	120
Algae	CHCl_3_/CH_3_OH, 2 : 1 (v/v), phase separation and evaporation to dryness; (2) H_2_O and evaporation to dryness; (3) 2% HNO_3_	Shaking/mixing + heating	HPLC-ICP-MS	39
Algae	20 mM ammonium acetate buffer (pH 7.4)	Sonication	HPLC-ICP-MS	141
Molluscs	(1) CH_3_OH/H_2_O, 1 : 1 (v/v)	Heating to 70 °C for 2 h in an oven then sonicated	HPLC-ICP-MS	18
	(2) 2% HNO_3_			
Algae, crustaceans, fish and molluscs	(a) CH_3_OH/H_2_O, 1 : 1 (v/v); (b) 2% HNO_3_	MAE-heating	HPLC-ICP-MS	96
Algae, crustaceans, fish and molluscs	2% HNO_3_	Shaking/mixing + sonication	HPLC-ICP/MS	142
Algae	1 M H_3_PO_4_ + 0.1 M ascorbic acid	MAE-heating	HPLC-HG-AFS	143

a HTLC: high temperature liquid chromatography, DMThioAsSugarGlycol: DMTAsGLY HPLC, high performance liquid chromatography; ICP-MS, inductively coupled plasma mass spectrometry; HG-AFS, hydride generation atomic fluorescence spectrometry; DRC: dynamic reaction cell.

## 7 Sample treatment

Extraction processes are occasionally selective. The initial extract often contains both the target analytes and co-extracted compounds, many of which can interfere with the analytical procedures. Consequently, thorough addressing of the matrix is essential to enhance the sensitivity and reliability of instrumental analysis, reduce interferences in chromatographic separation associated with the matrix, and improve analyte detection.^93^ One of the most useful techniques is utilizing silica gel clean approach. AsLipids are isolated from regular lipids in the sample matrix owing to their strong affinity for silica which interacts with acidic silica. As a consequence, AsLipids are firmly retained on the column, while normal lipids are eluted using solvents of low to moderate polarity. Because of having strong affinity of AsLipids for silica, high quantity of polar solvents such as MeOH are necessary to remove them.^144^ Methanol/dichloromethane extracts of marine algae were purified using silica gel in order to enhance chromatographic separation. This study demonstrated the effectivity of this technique for purifying arsenic-containing hydrocarbons (AsHCs) and arsenosugar phospholipids (AsSugar-PL) with seemingly insignificant loss.^145^ Nevertheless, when the method was applied to lipids in fish oil containing enormous quantities of As fatty acid (AsFA) conjugates, a significant quantity of the initial compounds were changed during the procedure.^146^

Solid-Phase Extraction (SPE) can be used to isolate interferent As^III^ on AsB in As speciation by incorporating an anionic cartridge ahead of the separation column. This manner results in the trap of As^III^, As^V^, MMA, and DMA.^147^ Finally, the acidic As-sugars were isolated from As-sugar-OH using size exclusion chromatography. This separation might occur because of the electrostatic repulsion between the anionic stationary phase and the anionic functional groups of the As-sugars causing the As-sugars to be liberated from the pores.^93,148^

## 8 Methods used for separation of arsenicals

There is a necessary to separate these arsenical efficiently in real samples.

### 8.1 Non-chromatographic method

#### 8.1.1 Capillary electrophoresis (CE)

Capillary electrophoresis (CE) emerges as an interesting technique for elemental speciation. Because of its excellent resolution, quick and effective separations, lowest reagent usage, chemical integrity and the ability to separate with minimal disturbance of the equilibrium between different species. It has been found that CE permits for the separation of different organic and inorganic As species.^149^ Owing to its exceptional efficiency of separation and comparatively mild separation conditions, which help in pressuring the initial form of As species in samples, CE affords a substitute separation method to high-performance liquid chromatography (HPLC). However, analysis of real sample because due to matrix interferences^150^ and the coupling of CE and inductively coupled plasma mass spectrometry (ICP-MS) demonstrates a challenging design hindrance, due to buffer incompatibilities with the ionization process.^151^ Although it has a relatively poor sensitivity, an on-column preconcentration technique with a column-switching facility has been incorporated into CE in order to improve sensitivity of arsenical determination.^152^ A combination of the advantages of CZE with mass spectrometry (MS) permits for the measurement of high separation efficiency and molecular masses and/or fragmentation in a single analysis. This has significant implications for the speciation of As in marine specimens.^153^

### 8.2 Chromatographic methods

#### 8.2.1 Gas chromatography

Gas chromatography (GC-MS) has successfully been developed for the measurement of As-containing hydrocarbons in marine sample including fish oil, seaweed samples,^116^ capelin oil.^154^ GC-ICP-MS was used to measure As-containing hydrocarbons in cannedcod liver^58^ and commercial fish oils.^155^ Gas chromatography (GC) can exhibit highly effective separation of volatile As species addressing the difficulties linked with the introduction of organic solvents to ICP-MS in the context of reversed-phase high-performance liquid chromatography (RP-HPLC). Nevertheless, the plasma of ICP-MS persists undisturbed by organic solvents when using GC. Moreover, GC is frequently enforced for the separation of fatty acids and other lipids that can be volatilized through derivatization. Despite these advantages, applications of GC-MS application in As speciation analysis is limited and few number of studies have focused on the application of GC for the analysis of arsenicals in lipophilic marine samples due to the non-volatile and thermolabile characteristic of most organoarsenicals.^88^

#### 8.2.2 Liquid chromatography

Water-soluble arsenicals with hydrophilic properties present in different ionic states that are pH-dependent. Devising a single scheme capable of separating all water-soluble As species is challenging because of variability of their hydrophilic properties.^156^ The presence of different alkyl groups make lipophilic As species mostly neutral with hydrophobic characteristics.^157^ Because of having differences in their physicochemical properties, different chromatographic methods have been documented to contribute in the speciation of As species in samples of marine extracts including ion-pairing reversed-phase, ion-exchange, ion-exclusion, and reversed-phase chromatographies.

##### 8.2.2.1 Ion exchange chromatography

The mechanism of exchange equilibria between a stationary phase, containing surface ions, and oppositely charged ions in the mobile phase is the base of ion exchange chromatograph (IEC) that has been applied to separate ionic and ionizable arsenicals. Two separation modes of IEC are available which includes anion and cation exchange chromatography.^158^

When the stationary phase is positively charged, and negatively charged molecules are injected to be retained to it (*i.e.*, the pH for chromatography is greater than the isoelectric point). This phenomena is named as anion-exchange chromatography. Due to differences in their anionic nature, anion IEC emerges as a viable alternative for separating these prevalent As species. As^V^ elutes slowly and has the highest negative charge in most mobile phases because of its low pKa1 (2.19) and pKa2 (6.98) values. Owing to high pKa values As^V^ elutes slowly and has the highest negative charge in most mobile phases because of its low pKa1 (2.19) and pKa2 (6.98) values, As^III^ is more likely to behave as a neutral molecule which elutes straightforward. Anion exchange chromatography has been applied to separate many As species such as As^III^, As^V^, MMA, DMA, AsB, AsC, oxo-As-sugars (oxoAsS), thio-As-sugars (thioAsS), and phenylarsenicals. Strong anion exchange column like PRP-X100 is the potential choice of column for separation of As species investigations.^159^

Positively charged As molecules such as AsB, AsC, TMAO, and TMA, have been frequently separated using cation exchange chromatography. Correspondingly to anion exchange, cation exchange chromatography functions by interacting with cationic analytes as a consequence the employing of a negatively charged stationary phase. Analytes that have stronger positively-charged retain in the column more than the weaker corresponding charges.^160^ Finally, there are many factors affect the performance of separation and retention of analytes in ion-exchange chromatography such as the ionic strength of the solute, the pH of the mobile phase, the ionic strength and concentration of the buffer, the temperature, the flow rate, and the addition of organic modifiers to the mobile phase.^161^

##### 8.2.2.2 Ion pair chromatography

In Ion-pair chromatography (IPC) makes use of aqueous solutions as the mobile phase, which may also include some organic modifiers to help with the separation of analytes on the less polar stationary phase. IPC has periodically been engaged in As speciation since it can differentiate between ionic and neutral species.^156^ In the IPC approach, ion pair reagents are introduced into the mobile phase of a standard reversed-column (C18). The charged group of the ion pair reagent interacts with the analyte while its hydrophobic portion interacts with the stationary phase. For the purpose of speciating anionic and neutral ions, as ion pair reagents tetraethylammonium, tetra-butylammonium, and tetraalkylammonium are commonly emplued as species. The separation of cationic and neutral ion pairs alkyl sulfonates, such hexane sulfonic acid and 1-pentane sulfonic acid, are widely used by species.^156^ In the mobile phase, ion-pair reagents are normally kept at low concentrations.^161^ However, there are challenges associated with using these counterions such non-selectivity and they also pair with matrix components hence they change the retention times.^162^ Acetonitrile and methanol are the two most frequently devoted organic modifiers. They are usually incorporated into the mobile phase which decrease retention time and change selectivity. While the neutral As species can interact with the conventional stationary phase directly, the charged As species must move through the hydrophobic stationary phase and the ion pair reagents. As^V^, As^III^, MMA, DMA, AsB, TMAO, TMA, and AsC have been separated using a mixed ion pair method with sodium butanesulfonate and tetramethylammonium hydroxide as the ion pairing reagents.^163^ The hydrophobicity of the counter-ion, the ion-pair reagent concentration, the buffer concentration, the pH and ionic strength of the mobile phase, and the characteristics of the stationary phase are some of the factors that affect the selectivity of chromatographic separation of analytes in ion-pair chromatography.^156^

##### 8.2.2.3 Reversed-phase-liquid chromatography

Reversed-phase (RP) liquid chromatography is very effective in the analysis of arsenolipids, which include fatty acids, phospholipids, phosphatidylcholines, fatty alcohols, and phosphatidylethanolamines of various kinds. Using ordinary C18 or C8 columns, arsenolipids can be separated based on the magnitude of double bonds, number of carbons, and other functional groups. RP-HPLC has been coupled with various detection instruments such as ICP-MS and ESI-MS to quantify and identify numerous arsenolipids in fish,^57^ macroalgae^81,164^ cod liver oil,^165^ capelin oil,^166^ fish meal from capelin and cod liver.^167,168^

IP-RP-HPLC has been employed to separate As^III^, AsB, DMA, and an arsenosugar (oxo-arsenosugar-glycerol, As 328) in extracts of commercial kelp and bladderwrack. This method has coupled to particle beam-electron ionization mass spectrometry (PB-EIMS).^169^ The stability of As fatty acids AsFA-362 and AsFA-388 which are arsenolipids was examined concerning sample storage and transport, as well as their preparation for quantitative analyses.^164^ RP chromatography is more sensitive to matrix and pH effects.^170^

##### 8.2.2.4 Size exclusion chromatography

Size exclusion chromatography (SEC) is not powerful for speciation of tiny As components. This is due to the size differences between many small As species and cannot be separated on an SEC column. Therefore, this technique is especially practical when As bonds with big components.^171,172^ A unique technique was created based on size exclusion chromatography connected to electrospray ionization mass spectrometry (SEC-ESI-MS) with the goal of preserving the intact proteins and their As bindings. SEC-ESI-MS was employed to measure the simultaneous interacting of phenylarsine oxide to five distinct peptides and proteins (glutathione, oxytocin, aprotinin, lactalbumin, and thioredoxin) were examined.^173^ The As-biomolecule complexes in *Mus musculus* liver extracts was measured using SEC.^174^

##### 8.2.2.5 High temperature liquid chromatography (HTLC)

High temperature liquid chromatography (HTLC) was coupled for analysis of oxo-As-sugars in extracts from marine organisms. Using plain water as the mobile phase—which has advantages over organic or saline mobile phases in terms of waste reduction, lack of salt deposition at the ICPMS interface, and matrix effects—the separation was carried out at 120 °C on a graphite column (Thermo Hypercarb).^125^

##### 8.2.2.6 Hydrophilic interaction liquid chromatography

Hydrophilic interaction liquid chromatography (HILIC) was created for the separation of the main conversion products. Therefore, affinity of the employed solvents for sepeciation analysis with both ICP-MS and ESI-MS was achieved. High-resolution electrospray (EC)-HILIC-ICP-MS was developed to measure the amount of the composed products, identify the original commodity evolved and quantification of As-containing species. HILIC was utilized to separate thirteen As species. This technique was coupled to EC-ICP-MS in a research of roxarsone electrochemical transformation products.^175^ A zwitterionic HILIC column was amenable to separate nine organoarsenicals successfully (*i.e.*, 3-nitro-4-hydroxyphenylarsonic acid (roxarsone, Rox), phenylarsonic acid (PAA), *p*-arsanilic acid (*p*-AsA), phenylarsine oxide (PAO), DMA, MMA, AsB, AsC and TMAO within 45 min, then coupled to ICP-MS/ESI-MS for detection these species.^176^

##### 8.2.2.7 Micellar liquid chromatography

Micellar Liquid Chromatography (MLC) has many advantages including coetaneous separation of both ionic and non-ionic analytes, faster analysis times, and enhanced detection sensitivity and selectivity.^177^ These results from its special three-way equilibrium mechanism, in which, in addition to the mobile and stationary phases, micelles also function as a pseudo-phase. This approach has been used in As speciation.^178^

##### 8.2.2.8 Multifaceted chromatographic techniques

Because of the existence of a range of As species particularly in matrix like seafood, combination of different chromatographic approaches has also been applied. This arise from the total separation of As species with a decrease in co-elution possibilities, improving the accuracy of analytical outputs.^93,179^ The use of two or more chromatogaraphic separation method has several benefits such as improving the effectiveness of chromatographic separation and, in particular, aid in achieving baseline resolution.^180,181^ For the simultaneous separation of neutral and ionic species, reversed-phase ion-pairing chromatography is a perfect substitute. To improve the retention capacity of As species on a C_18_ column, chelating agents such as sodium 1-butanesulfonate, malonic acid, and TMAH have been employed in conjunction with a variety of alkyl sulfonates as anion pair reagents.^98^

## 9 Detection techniques for As speciation

Different techniques have been reported for analysis of As involving atomic spectroscopy and molecular mass spectrometry that can identify an element specifically. Among these, one of the most valuable and versatile technique is inductively coupled plasma mass spectrometry (ICP-MS). Detection techniques such as s inductively coupled plasma optical emission spectrometry (ICP-OES), atomic absorption spectrometry (AAS), atomic fluorescence spectrometry (AFS), or electrothermal atomic absorption spectrometry (ET-AAS) have also been utilized.^25,182^ Literature of As species measured in marine samples using different detection techniques worldwide are presented in Table 2.

**Table 2:** Commonly utilized coupling techniques for detecting As species in various marine organisms in the literature^a^

Sample matrix	Technique	Contents of As species (μg g^−1^)	Separation condition	Detection limit ng mL^−1^	Reference
LC-ICP-MS/MS	Shark	AsB:13.9, As^III^: < 0.03, DMA: <0.006, unknown: <0.012, MMA: <0.012, As^V^: <0.026	PRP-X100 (250 × 4.1 mm, 10 μm); PRP-X100 (20 × 2 mm, 10 μm); 10 mmol L^−1^ (NH_4_)_2_HPO_4_ diluted in 1% (v/v) methanol (pH 8.65); 50 μL	AsB: 6, As^III^: 30, DMA: 12, MMA, As^V^, 26 ng g^−1^ (LOQ)	140
	Shrimp	AsB: 12.06, As^III^: <0.03, DMA: <0.006, unknown: <0.012, MMA: <0.012, As^V^: <0.026			
	Squid	AsB: 1.31, As^III^: <0.03 DMA: <0.006, unknown: <0.012, MMA: <0.012, As^V^: 0.14			
	Oyster	AsB: 5.0, As^III^: 0.26, DMA: 0.7, unknown: 0.41, MMA: <0.012, As^V^: <0.026			
	Scallop	AsB: 0.58, As^III^: <0.03, DMA: <0.006, unknown: <0.012, MMA: <0.012, As^V^: 0.15			
HPLC-ICP-MS	Ling	AsB: 17.99 ± 1.5, DMA: <LOD, MMA: 0.18 ± 0.01, As^V^: 0.42 ± 0.01	Hamilton resin PRP-X100, 10 μm particle size, sulphate (Na_2_SO_4_), ammonium dihydrogen phosphate (NH_4_H_2_PO_4_), phosphate (NH_4_H_2_PO_4_) mobile phases (isocratic elution) *A* = 6.5 mmol L^−1^ Na_2_SO_4_, pH 10.2, 5% CH_3_OH *B* = 20 mmol L^−1^ NH_4_H_2_PO_4_, pH 6.0, 1% CH_3_OH	AsB: 0.015, DMA: 0.022, MMA: 0.034, As^V^: 0.027	87
	Gurnard	AsB: 11.98 ± 0.18, DMA: <LOD, MMA: 0.53 ± 0.01, As^V^: 0.19 ± 0.01			
	Grey mullet	AsB: 3.41 ± 0.15, DMA: 0.46 ± 0.02 MMA: 0.32 ± 0.02, As^V^: 0.60 ± 0.03			
	Pollock	AsB: 23 ± 0.59 DMA: <LOD MMA: 0.61 ± 0.04, As^V^: 0.22 ± 0.01			
	Dover sole	AsB: 51.18 ± 4.77, DMA: 0.1 ± 0.01 MMA: 0.58 ± 0.07, As^V^: 0.30 ± 0.01			
	John dory	AsB: 3.60 ± 0.12, DMA: 0.25 ± 0.02 MMA: <LOD, As^V^: <LOD			
	Megrim	AsB: 26.47 ± 1.44, DMA:<LOD, MMA: 0.27 ± 0.03, As^V^: 0.55 ± 0.02			
	Flounder	AsB: 25.64 ± 1.92, DMA: 0.16 ± 0.01, MMA: 0.57 ± 0.02, As^V^: 0.90 ± 0.11			
	Dab	AsB: 51.20 ± 2.27, DMA: <LOD, MMA: 0.30 ± 0.02, As^V^: 0.74 ± 0.02			
	Sand sole	AsB: 29.37 ± 2.91, DMA: <LOD, MMA: 0.63 ± 0.01, As^V^: 1.09 ± 0.03			
	Brill	AsB: 13.07 ± 0.69, DMA: <LOD, MMA: 0.61 ± 0.02, As^V^: 0.403 ± 0.02			
	Lemon sole	AsB: 74.09 ± 3.57, DMA: 0.13 ± 0.01, MMA: 0.24 ± 0.02, As^V^: 0.5 ± 0.04			
	Halibut	AsB: 97.74 ± 5.20, DMA: <LOD, MMA: 0.40 ± 0.03, As^V^: 0.64 ± 0.04			
HPLC-ICP-MS	Clam	DMA: 0.15–0.41, AsB: 6.7–67, As-Gly; 0.33–2.5, TETRA: <LOD-2.2	Hamilton PRP-X100 column (4.6 × 150 mm, 5 μm) at 40 °C with malonic acid buffers	0.01	126
	Oyster	DMA: 0.11–0.27, AsB: 25–64, As-Gly: 0.14–0.66, TETRA: <LOD			
	*Arius thalassinus*	AsB: 46–66, As-Gly: <LOD, TETRA: <LOD			
	*Sepia pharaonis* (cuttlefish)	AsB: 61–114, As-Gly: <LOD, TETRA: <LOD			
	*Parupeneus margaritatus*	AsB: 39–53, As-Gly:<LOD, TETRA: <LOD			
	*Acanthopagrus bifasciatus*	AsB: 14–74, As-Gly: 0.49–0.7, TETRA: <LOD			
	*Rhabdosargus haffara*	AsB: 16–20, As-Gly: <LOD, TETRA: <LOD			
	*Penaeus semisulcatus* (shrimp)	AsB: 27, As-Gly: <LOD, TETRA: 0.65			
	*Carangoides fulvogutatus*	AsB: 45–51, As-Gly: <LOD, TETRA: <LOD			
	*Nemipterus japonicas*	AsB: 26–32, As-Gly: <LOD-0.27, TETRA: <LOD			
	*Argyrops spinifer*	AsB: 8.2, As-Gly: <LOD, TETRA: <LOD			
HPLC-ICP-MS	*Lobophora* sp.	AsB: 0.69 ± 0.1, OH-R: 0.43 ± 0.06, T-SO_3_: 0.06 ± 0.01, T-PO_4_: ND, TOSO_3_: ND, T-Gly: ND	PRP-X100 (150 mm × 21 mm, 12–20 μm) using ammonium carbonate buffer, pH 10	0.01	114
	*Sargassum* sp.	AsB: ND OH-R: 4.9 ± 0.7, T-SO_3_: ND, T-PO_4_: ND, TOSO_3_: ND, T-Gly: ND			
	*Hormosira banksia*	AsB: ND, OH-R: 6.2 ± 0.9, T-SO_3_: ND, T-PO_4_: ND, TOSO_3_:ND, T-Gly: ND			
	*Ascophyllum nodosum*	AsB: ND, OH-R: 8.5 ± 1.3, T-SO_3_: 0.17 ± 0.03, T-PO_4_: 0.13 ± 0.02, TOSO_3_: ND, T-Gly: ND			
	*Ecklonia radiate*	AsB: ND, OH-R: 1.9 ± 0.3, T-SO_3_: ND, T-PO_4_: ND, TOSO_3_: ND, T-Gly: ND			
	*Macrocystis pyrifera*	AsB: ND, OH-R: 23 ± 4, T-SO_3_: ND, T-PO_4_: ND, TOSO_3_: ND, T-Gly: ND			
	*Padina fraseri*	AsB: 0.50 ± 0.08, OH-R: 0.39 ± 0.06, T-SO_3_: ND, T-PO_4_: ND, TOSO_3_: ND, T-Gly: ND			
	*Durvillaea potatorum*	AsB: ND, OH-R: 14 ± 2, T-SO_3_: ND, T-PO_4_: ND, TOSO_3_: ND, T-Gly: ND			
	*Amphiroa anceps*	AsB: 0.23 ± 0.03, OH-R: 0.44 ± 0.07, T-SO_3_: ND, T-PO_4_: ND, TOSO_3_: 0.027 ± 0.004, T-Gly: ND			
	*Martensia fragilis*	AsB: 0.15 ± 0.02, OH-R: 0.12 ± 0.02, T-SO_3_: ND, T-PO_4_: ND, TOSO_3_: ND, T-Gly: ND			
	*Laurencia* sp.	AsB: 0.56 ± 0.08, OH-R: 2.8 ± 0.4, T-SO_3_: 0.16 ± 0.02, T-PO_4_: ND, TOSO_3_: ND, T-Gly: ND			
	*Corallina officinalis*	AsB: ND, OH-R: 0.23 ± 0.03, T-SO_3_: ND, T-PO_4_: ND, TOSO_3_: 0.027 ± 0.004, T-Gly: ND			
	*Codium lucasii*	AsB: 0.71 ± 0.11, OH-R: 1.1 ± 0.2, T-SO_3_: ND, T-PO4: ND, TOSO_3_: ND, T-Gly: ND			
	*Cladophora subsimplex*	AsB: 1.2 ± 0.2, OH-R: 3.2 ± 0.5, T-SO_3_: ND, T-PO_4_: ND, TOSO_3_: ND, T-Gly: ND			
HPLC-ICP-MS	*C. rodgersii* (visceral)	AsB: 7.9 ± 0.4, OH-R: 0.93 ± 0.04, TriMeOH: <0.01, TMAP: <0.01, DMAE: 1.1 ± 0.1, AC: <0.01, TETRA: <0.01	PRP-X100 (150 mm × 21 mm, 12–20 μm) using ammonium carbonate buffer, pH 10	0.01	114
	*C. rodgersii* (gonad)	AsB: 3.9 ± 0.2, OH-R: 0.51 ± 0.01, TriMeOH: <0.01, TMAP: 0.19 ± 0.01, DMAE: 1.1 ± 0.1, AC: 0.03 ± 0.01, TETRA: 0.06 ± 0.01			
	*O. cyanomelas* (food pellets)	AsB: 0.58 ± 0.03, OH-R: 0.08 ± 0.01, TriMeOH: <0.01, TMAP: <0.01, DMAE: 0.31 ± 0.01, AC: <0.01, TETRA: <0.01			
	*O. cyanomelas* (muscle)	AsB: 0.15 ± 0.01, OH-R: 0.14 ± 0.01, TriMeOH: <0.01, TMAP: 0.07 ± 0.01, DMAE: <0.01, AC: <0.01, TETRA: <0.08 ± 0.01			
	*O. cyanomelas* (liver)	AsB: 0.50 ± 0.02, OH-R: 0.26 ± 0.01, TriMeOH: <0.01, TMAP: <0.01, DMAE: <0.01, AC: 0.04 ± 0.01, TETRA: <0.01			
	*O. cyanomelas* (digestive)	AsB: 0.21 ± 0.01, OH-R: 1.6 ± 0.1, TriMeOH: <0.01, TMAP: <0.01, DMAE: <0.01, AC: 0.04 ± 0.01, TETRA: 0.05 ± 0.01			
	*O. cyanomelas* (gill)	AsB: 0.96 ± 0.05, OH-R: 0.66 ± 0.03, TriMeOH: <0.01, TMAP: <0.01, DMAE: <0.01, AC: <0.01, TETRA: <0.01			
	*O. cyanomelas* (gut contents)	AsB: <0.01, OH-R: 2.6 ± 0.1, TriMeOH:<0.01, TMAP: <0.01, DMAE: <0.01, AC: <0.01, TETRA: <0.01			
HPLC-UV-HG-AFS	Pelagic fish	AsB: 1.22–5.23, As^III^: 0.01–0.08, MMA: <LOD-0.1, DMA: 0.02–0.45, As^V^: <LOD-0.11	Mobile phases (NH_4_HCO_3_ and KCl) and HCl and KOH employed for hydride generations	NG	127
	Benthic fish	AsB: 2.79–28.9, As^III^: 0.01–0.07, MMA: <LOD-0.1, DMA: 0.01–0.26, As^V^: <0.02–0.17			
	Shrimp	AsB: 8.58–29.9, As^III^: 0.01–0.05, MMA: <LOD-0.03, DMA: 0.02–0.08, As^V^: <0.02–0.11			
	Crab	AsB: 12.7–37.7, As^III^: 0.01–0.11, MMA: <LOD-0.10, DMA: 0.03–0.15, As^V^: <0.02–0.12			
HPLC/ICP-DRC-MS	Silver bream	AsB: 0.0752–0.088, As^V^ < LOD	Anion-exchange column PRP-X100 (4.6 mm × 150 mm) 10 mmol L^−1^ of ammonium dihydrogen phosphate and 10 mmol L^−1^ of ammonium nitrate	0.056 for total As to 0.15 for As^V^	25
	Bream	AsB: 0.3008–0.447, As^V^: <LOD-0.0101			
	Trout	AsB: 3.87–4.16, As^V^: 0.0570–0.1337			
	Sturgeon	AsB: 5.23, As^V^; 0.0379			
	Carp	AsB: 0.0604, As^V^; <LOD			
LC-ICP-MS	Cod	As^III^: ND, As^V^: 0.0049, AsB: 1.2, DMA: 0.017, DMAA: ND, DMAP: ND, DMAE: ND, MMA: ND, TMAO: ND, TMAP: 0.0039, AC: 0.0031, TMA: 0.0014, Sug328: ND, Sug392: ND, Sug408: ND, Sug482: ND	PRP-X100, Hamilton, mobile phase: A: 5 mM NH_4_HCO_3_, B: 50 mM (NH_4_)_2_CO_3_	As^III^: 1.1, As^V^: 2, AsB: 0.9, DMA: 1.2, DMAA: 1, DMAP: 1, DMAE: 1.5, MMA: 0.8, TMAO: 1, TMAP: 1.5, AC: 0.8, TMA: 1.2, Sug328: 1, Sug392: 1.1, Sug408: 2, Sug482: 1.8 (ng g^−1^)	120
	Haddock	As^III^: ND, As^V^: 0.005, AsB: 6, DMA: 0.014, DMAA: ND, DMAP: ND, DMAE: 0.014, MMA: ND, TMAO: ND, TMAP: 0.0108, AC: 0.0078, TMA: 0.033, Sug328: ND, Sug392: ND, Sug408: ND, Sug482: ND			
	Mackerel	As^III^: 0.0014, As^V^: 0.0066, AsB: 0.405, DMA: 0.039, DMAA: ND, DMAP: 0.0043, DMAE: ND, MMA: 0.0012, TMAO: 0.0072, TMAP: 0.0085, AC: 0.0062, TMA: 0.0037, Sug328: ND, Sug392: ND, Sug408: ND, Sug482: ND			
	Crab	As^III^: ND, As^V^: 0.0076, AsB: 21, DMA: 0.0078, DMAA: 0.002, DMAP: ND, DMAE: 0.015, MMA: 0.0045, TMAO: 0.0033, TMAP: 0.052, AC: 0.0052, TMA: 0.011, Sug328: 0.02, Sug392: ND, Sug408: 0.0065, Sug482: 0.0056			
	Shrimp	As^III^: ND, As^V^: 0.0078, AsB: 0.115, DMA: ND, DMAA: ND, DMAP: ND, DMAE: ND, MMA: ND, TMAO: ND, TMAP: 0.0042, AC: ND, TMA: ND, Sug328: ND, Sug392: ND, Sug408: ND, Sug482: ND			
	Geoduck	As^III^: ND, As^V^: 0.015, AsB: 0.323, DMA: 0.026, DMAA: ND, DMAP: 0.0032, DMAE: 0.019, MMA: ND, TMAO: ND, TMAP: ND, AC: 0.0095, TMA: ND, Sug328: 0.247, Sug392: ND, Sug408: 0.0059, Sug482: 0.529			
	Oyster	As^III^: 0.0056, As^V^: 0.0089, AsB: 0.554, DMA: 0.058, DMAA: ND, DMAP: 0.0039, DMAE: ND, MMA: 0.0019, TMAO: ND, TMAP: 0.0074, AC: 0.0052, TMA: ND, Sug328: 0.038, Sug392: ND, Sug408: ND, Sug482: 0.328			
	Kombu	As^III^: 0.027, As^V^: 0.322, AsB: 0.352, DMA: 0.427, DMAA: ND, DMAP: 0.023, DMAE: ND, MMA: ND, TMAO: 0.0018, TMAP: 0.019, AC: 0.012, TMA: 0.012, Sug328: 1.876, Sug392: 2.37, Sug408: 7.615, Sug482: 1.234			
HPLC-ICPMS/ES-MS	Dulse	C18H36O3As: <LOD-0.017, C20H44OAs: 0.048–0.052, C47H89O14AsP: 0.01–0.032, C45H89O14AsP: <LOQ-0.015 (AsLipd)	Reverse-phase column (Agilent Eclipse XDB-C18; 4.6–150 mm) with a gradient of water and methanol both in 0.1% formic acid was used for the speciation of AsLps	0.003 (mg kg^−1^)	218
HPLC-ICPMS/ES-MS	Capelin (*Mallotus villosus*)	C_17_H_36_AsO_3_: 0.0014, C_23_H_38_AsO_3_: 0.0030, C_24_H_38_AsO_3_: 0.0047, C_23_H_38_AsO: 0.061, C_17_H_38_AsO: 0.175, C_19_H_42_AsO: 0.081	Column of ACE C18; 4.6 mm 150 mm, 5 μm, mobile phase composed of buffer: 0.1% formic acid in water buffer, B: 0.1% formic acid in methanol	0.01	59
HPLC-ICP-MS/ESI-Q-TOF-MS	Herring	AsB: 11.0, As^III^: 0.021, DMA: 0.103, As^V^: <0.09	Column: Atlantis C18 (5 mm, 4.6 150 mm, waters), mobile phase: eluent A: 0.1% formic acid in water eluent, B: 0.1% formic acid in methanol	NG	43
	Salmon	AsB: 47, As^III^: 0.020, DMA: <0.02, As^V^:<0.09			
	Mackerel	AsB: 50, As^III^: 0.020, DMA: <0.020, As^V^: <0.09			
	Pilchard	AsB: 63, As^III^: 0.013, DMA: 0.098, As^V^: LOD			
	Wolffish	AsB: 87, As^III^: 0.131, DMA: LOD, As^V^: <0.09			
	Cape hake	AsB: 81, As^III^: 0.007, DMA: 0.063, As^V^: <0.09			
	Plaice	AsB: 78, As^III^: 0.039, DMA: <LOD, As^V^: LOD			
HPLC-ICP-MS/ESI-MS	Wakame	As-HC332: 0.022, As-HC360: 0.082, As-HC388: 0.406, As-PL958: 0.426, As-PL988: 0.144, As-PL956: 0.200, As-PL1014: 0.226, As-PL1042: 0.027, As-PL1070: ND	Zorbax Eclipse XDB-C8 column (4.6–150 mm; 5 mm particle size) and a mobile phase comprising acetic acid (10 mmol L^−1^ at pH 6.0, adjusted with aqueous ammonia) and methanol	NG	53
	Hijiki	As-HC332: 0.309, As-HC360: 0.035, As-HC388: 0.022, As-PL958: 0.251, As-PL988: 0.085, As-PL956: 0.058, As-PL1014: 0.048, As-PL1042: 0.032, As-PL1070: 0.021			
HPLC-ICPMS	*Anadonta anatine*	AsB: <LOD-0.0461, DMA: 0.0354–0.0414, As^V^: 0.0259–0.0443, Gly-sug: 0.145–0.353, phosphate sugar: 0.210–0.273, thio Gly-sug: 0.0994–0.142, thio phosphate sugar: 0.142–0.158	Cation-exchange: ZORBAX 300-SCX (15 cm × 4.6 mm, 5 mm), mobile phase: 10 mM pyridine, pH: 2.6	10 μg As kg^−1^	136
	*Dreissena polymorpha*	AsB: <LOD, DMA: 0.0396, As^V^: 0.0693, Gly-sug: 0.194, phosphate sugar: 0.182, thio Gly-sug: 0.0799–0.142, thio phosphate sugar: 0.0898	Anion exchange: PRP-X100 (25 cm × 4.1 mm, 10 mm), mobile phase: 20 mM NH_4_H_2_PO_4_, pH: 5.6		
	*Sinanadonta woodiana*	AsB: <LOD, DMA: 0.0411–0.0966, As^V^: trace-0.0113, Gly-sug: 0.0725–0.192, phosphate sugar: 0.180–0.404, thio Gly-sug: 0.025–0.0581, thio phosphate sugar: 0.0869–0.133	Anion-exchange: PRP-X100 (10 cm × 4.1 mm, 5 mm), mobile phase: 20 mM NH_4_HCO_3_, pH:10.3		
	*Unio pictorum*	AsB: Trace-<LOD, DMA: 0.0525–0.0932, As^V^: Trace-0.0185, Gly-sug: 0.323–0.701, phosphate sugar: 0.247–0.614, thio Gly-sug: 0.0917–0.195, thio phosphate sugar: 0.0855–0.235			
HPLC-ICPMS	Gastropods gut	As^III^: <LOD-3.2, DMA: 0.12–0.17, MMA: <LOD-0.13, As^V^: <LOD-2.2, PO_4_-sug: <LOD, AsB: 1.78–24.9, AC: <LOD-0.42, TMAP: 0.15–0.51, TETRA: 0.25–1.69, Gly-sug: 0.27–0.61	Anion-exchange: Hamilton PRP-X100 (250 × 4.1 mm, 10 μm), mobile phase: 20 mM NH_4_H_2_PO_4_, pH = 5.8	8 to 155	219
	Gastropods tissue	As^III^: <LOD-2.9, DMA: <LOD, MMA: <LOD, As^V^: <LOD-0.17, PO_4_-sug: <LOD, AsB: 2.8–30.5, AC: <LOD-0.18, TMAP: 0.33–1.2, TETRA: 0.16–2.4, Gly-sug: <LOD	Cation-exchange: Zorbax 300-SCX (250 mm × 4.6 mm, 5 μm), mobile phase: 20 mM pyridine, pH = 2.6		
HPLC-ICPMS	*Crustacean* calappa sp.	AsB: 4.20, AC: <0.01, TETRA: 0.05, TMAO: 0.02, DMA: 0.06, MMA: <0.005, As^III^: <0.01, As^V^: 0.02	An inertsil AS column (15 cm × 2.1 mm i.d.) was used the column was equilibrated with the mobile phases (10 mM sodium 1-butanesulfonate, 4 mM tetramethylammonium hydroxide, 4 mM malonic acid and 0.5% methanol, pH 3.0 was adjusted with nitric acid) at a flow rate of 0.5 mL min^−1^ at 45 °C	0.005–0.01	139
	*Portunus trituberculatus*	AsB: 0.81, AC: <0.01, TETRA: 0.07, TMAO: 0.01, DMA: 0.01, MMA: <0.005, As^III^: <0.01, As^V^: <0.01			
	*Charybdis* sp.	AsB: 4.55, AC: 0.01, TETRA: 0.10, TMAO: 0.01, DMA: 0.04, MMA: <0.005, As^III^: <0.01, As^V^: 0.01			
	*Penaeus monodon*	AsB: 7.96, AC: 0.02, TETRA: 0.17, TMAO: 0.01, DMA: 0.04, MMA: 0.014, As^III^: 0.03, As^V^: 0.08			
	*Penaeus merguiensis*	AsB: 0.74, AC: <0.01, TETRA: 0.10, TMAO: 0.01, DMA: 0.06, MMA: <0.005, As^III^: <0.01, As^V^: <0.01			
	*Cephalopod* Octopus sp	AsB: 3.92, AC: 0.05, TETRA: 0.06, TMAO:0.01, DMA: 0.06, MMA: <0.005, As^III^: 0.01, As^V^: 0.03			
	*Acanthogobius lavimanus*	AsB: 0.65, AC: <0.01, TETRA: 0.04, TMAO: <0.01, DMA: 0.01, MMA: 0.016, As^III^: <0.01, As^V^: <0.01			
	*Plotosus canius*	AsB: 3.01, AC: <0.01, TETRA: 0.01, TMAO: 0.04, DMA: 0.05, MMA: 0.007, As^III^: 0.01, As^V^: 0.07			
	*Sillago sihama*	AsB: 1.93, AC: <0.01, TETRA: 0.03, TMAO: <0.01, DMA: <0.01, MMA: 0.009, As^III^: 0.01, As^V^: 0.06			
	*Oreochromis niloticus*	AsB: 1.27, AC: <0.01, TETRA: 0.03, TMAO: 0.01, DMA: 0.10, MMA: <0.005, As^III^: <0.01, As^V^: 0.01			
	*Scatophagus argus*	AsB: 0.41, AC: <0.01, TETRA: 0.01, TMAO: 0.09, DMA: 0.06, MMA: <0.005, As^III^: 0.01, As^V^: 0.04			
	*Lates calcarifer*	AsB: 0.91, AC: <0.01, TETRA: 0.03, TMAO: <0.01, DMA: 0.02, MMA: <0.005, As^III^: 0.02, As^V^: 0.03			
HPLC-ES-MS/MS	Kelp	DMThioAsSugarGlycol: NG, DMThioAsSugarPhosphate: 0.25, DMThioAsSugarSulfonate: 2.6, DMThioAsSugarSulfate: 3.3	Anion exchange chromatography (PRP-X100, 250–4.1 mm with two PRPX800 cation exchange pre-columns; Hamilton, Reno, NV) was applied for online HPLC-ES-MS/MS. Gradient elution A: 20 mM NH_4_HCO_3_, pH 10 and B: 20 mM NH_4_HCO_3_, 40% methanol, pH 10	NG	115

a LOD: limit of detection, OH-R: OH-riboside, T-SO_3_:thio-SO_3_ – riboside,T-PO_4_: thio-PO_4_ – riboside, TOSO_3_- Thio-OSO_3_-riboside, T-Gly: thio-gly-riboside, TriMeOH: glycerol trimethylarsonioriboside, TMAP: trimethylarsoniopropionate, DMAE: 2-dimethylarsinoyl, LOQ: limit of quantification of arsenolipid = 0.010 μg g^−1^, NG: not given, co-electro-osmotic flow (co-EOF) capillary zone electrophoresis (CZE), FF: flower and fruits, S: strawberry; values are less than limit of quantification; ETV: electrothermal vaporization.

### 9.1 AAS

Element-specific detection, minimized matrix effects, sensitivity, simplicity, and precision at low parts per billion levels have made AAS attractive and superior approach in detection of As species comparing with other techniques.^7,86,183^ In order to facilitate As analysis, hydrides must frequently be generated when employing AAS. Utilizing HG as sample introduction unit can offer special advantages for the measurement of As speciation, such as the ability to separate and enrich analytes from the matrix, introduce samples with high efficiency, and significantly reduce spectroscopic or matrix interferences from samples containing high concentrations of acid and salt.^98^ Fraction collection and online coupling of HPLC with GFAAS lead to improve sensitivity of the detection methods and reducing the detection limits to become in the range of a few nanograms.^7,183^ In addition, a rapid and efficient approach for simultaneous separation and measurement of different As species in marine products involves the use of HPLC in conjunction with HG-AAS or HG-AFS. This approach combines the effectiveness of post-column online derivatization, the distinctive gas–liquid separation techniques of chemical vapor generation, and the high separation efficiency of HPLC.^98^ A high-intensity boosted discharge hollow-cathode lamp was employed to improve the baseline noise level which leads to produce a lower detection limit of 0.26 ppb for a sample volume of 16 μL (equivalent to 4.2 pg. As).^184^

Because of its convenience, ease of use, and modest, AAS is a widely employed method for metal identification. To increase the metrological aspects of AAS, particularly sensitivity and detection limits, sample pretreatment is typically conducted prior to the actual detection stage.^185^ When coupled with various separation techniques and chemical modifiers, optical spectroscopy becomes a valuable tool for identifying As^III^, As^V^, DMA, MMA, AsC, AsB, and TAMO, as well as detecting significant hydride As-sugars and thioarsenate synthesis.^186,187^

Nonetheless, the chemical forms and valence states of the analytes influence the effectiveness of hydride generation (HG). Pentavalent As species undergo HG lesser readily than their trivalent counterpart, therefore detection sensitivity decreased. Moreover, the range of organoarsenic species has limited capability to generate hydrides using chemical reagents. Consequently, a chromatographic eluent is often subjected to UV radiation^157^ or microwave digestion^91^ are used to convert from inactive to active species before analysis using post-column derivatization.

### 9.2 Atomic fluorescence spectroscopy

Atomic fluorescence spectroscopy (AFS) method has proven to be vital alternatives to mass spectrometric methods in many applications. This is basically due to their low acquisition and purchases, low detection limits, reproducibility, rapid analysis warm-up interval of analysis repeatability and decreased matrix effects, especially when combined with hydride generation for As species.^90^ The use of HPLC in conjunction with atomic fluorescence spectrometry (AFS) for As speciation is now well-established and effective. AFS is a good substitute for other atomic spectrometers that are frequently used in speciation research, like AAS and ICP-MS.^87^ Regarding performance factors like detection limits, reproducibility, repeatability, and sensitivity for As, AFS can compete with ICP-MS.^188–191^ Moreover, HG-AFS is able to discriminate between As compounds that are hydride-active and those that are not, offering a precise measurement of the more hazardous species. While distinguished hydride generation has been noted for As-sugars and thioarsenates, it is generally acknowledged that the production of volatile analytes from As species is confined to As^III^, As^V^, MA, DMA, and TMAO. This complexity adds challenges to the interpretation of analytical data.^192^

### 9.3 Inductively coupled plasma mass spectrometry

Inductively coupled plasma mass spectrometry (ICP-MS) is currently implemented for As speciation in the majority of laboratories. With double-focusing sector field ICP-MS, target elements can be promptly measured without the obligation for isolation or pre-concentration. Additionally, it provides low LODs, high sensitivity across a broad linear dynamic range, and the capability for multi-element analysis.^193,194^ Due to its exceptional sensitivity, selectivity, rigorous isotopic estimation ratio (even though not for As), measurement of numerous elements at low content (LOD = 1–10 pg mL^−1^) and extensive dynamic range, ICP-MS is the most employed technique for As speciation. Different methods have been devised to mitigate or eliminate isobaric interferences—identical mass isotopes of various elements present in the same sample—to enable the identification of As at a mass-to-charge ratio of 75.^195^ ICP-MS is highly robust and provides lower susceptibility to impact of matrix. The high capability of sampling and acquiring data rates of ICP-MS permit for baseline separation of neighboring peaks, facilitating determination regardless of compromising peak resolution.^93,156^

#### 9.3.1 Signal interferences in ICP-MS

Single quadrupole mass spectrometers are not able to eliminate spectroscopic interference completely since they limited resolution capability of approximately 0.75 atomic mass unit (amu). Resolution is related to mass spectrometry data which refers to the mass spectrometer's capacity to discriminate between ions with varying mass-to-charge ratios (*m*/*z*). More resolution enables the instrument to distinguish between ions with nearly identical *m*/*z* values, improving the identification and measurement of the ions in a sample.^196^ This makes HPLC-ICP-MS sensitive to disruption by polyatomic ions that have the same mass-to-charge ratio (*m*/*z*) as the components being analyzed.^98^ Interferences in ICP-MS can occur when there is an isobaric overlap brought on by polyatomic ions created by the combining of two or more atoms. The most significant polyatomic ions are produced by a combination of the most prevalent argon isotopes, surrounding gases, and the solvents or acids employed in sample preparation.^7^^40^As^35^Cl is the most common polyatomic ion interference [As is monoisotope *m*/*z* 75] during As determination in the marine samples which ultimately affects the selectivity of ICP-MS.^195^

Interferences in ICP-MS can be reduced using various methods. ^40^As^35^Cl^+^ interference was quantitatively eliminated by improving chromatographic modes in such a way which As species eluted before chloride ions from the column.^197^ In addition, polyatomic interferences can be decreased by mixing a different gas to the argon plasma, such as N_2_, O_2_, air, He, or H_2_, which can also decreases the main polyatomic interference.^198^ For the removal of interferences, a more modern approach utilizing collision cell technology is already available on commercial instruments. For elemental speciation studies, sector field (SF)-ICP-MS may be the best choice because of its sensitivity and ability to resolve isobaric overlaps.^199^^40^Ar^35^Cl^+^ was reduced in study of As speciation employing a collision reaction cell with gases such H_2_, O_2_, NH_3_, CH_4_, NO, CO_2_, and C_2_H_4_.^200–202^ In cases where refractory oxides may produce due to incomplete dissociation or recombination, particularly in colder plasma regions such as the boundary layer around the sampler cone, polyatomic interferences can appear. To address these interferences, a collision/reaction cell can be used in ICP-MS, incorporating a collision gas (such as He) or a reaction gas (such as O_2_, H_2_, or CH_3_F), or a combination of two gases. The use of ICP with triple quadrupole tandem mass spectrometry (ICP-QQQ) shows beneficial in eliminating isobaric interferences, minimizing background noise, and enhancing selectivity compared to conventional single quadrupole ICP-MS.^195^

The major challenge in determination of AsLipids drives in the incompatibility between ICP-MS and organic solvents that are necessary for the extraction of these As species.^203,204^ Mobile phases that containing high organic level can hamper signal enhancement or extinguish even the plasma for As analysis, demanding the addition of oxygen to help in carbon removal on the sampling cones of the interface due to incomplete combustion. This may affect analytical performance, leading to either analyte loss or a reduction in signal intensity.^115,205^

These problems can be solved by using a composed interface, such as a cooled spray chamber, membrane desolvator, or post-column dilution, especially with microbore LC columns. Implementing techniques like low solvent flow, introducing oxygen to the plasma gas, or incorporating a post-column flow split helps overcome these issues.^115,206,207^

### 9.4 Electrospray ionization mass spectrometry (ESI-MS)

Due to abundant structural and molar mass information, the HPLC-ESI-MS with a “soft” electrospray ionization (ESI) technique perform as substitute detection method in speciation analysis.^208^ Coupling of the electrospray ionization technique with high-resolution mass spectrometry (such as triple quadrupole mass spectrometry), Q-TOF and orbitrap provides exceptional separation for co-eluted components in HPLC and can be utilized for the identification of unknown species.^57,209,210^ In last few years, As^III^, As^V^, MMA, DMA, TMAO, AsB, AsC, TETRA, As-sugars, arsenolipids, and many other As species have been measured using ESI-MS/MS. The main drawback of HPLC-ESI-MS as individual detection technique is sensitivity which is lower than ICP-MS.^211^

In the recent times, the combination of HPLC with ICP-MS and high-resolution ESI-MS is very effective for the identification and quantification of As species. The employ of ESI-MS in conjunction with HPLC-ICP-MS not only offers confirmation details for As compounds but also provides identification outcome for unknown compounds.^212^ An analytical method has been developed that combines anion exchange chromatography coupled with ICP-MS and ES-MS/MS. This method enables to measure up to nine As species in just 9 minutes. Both targets the analytes and unknown compounds are determined by ICP-MS with certain mass-to-charge ratios (*m*/*z* 329, *m*/*z* 483, *m*/*z* 437).^209^

### 9.5 X-ray spectroscopic techniques

X-ray spectroscopic is an effective method for total and As speciation in samples especially biological media that contain high concentration of As and less sample preparations as well as lack of necessity of extracting element species.^213^ XAS is generally classified into two regions: the X-ray absorption near edge structure (XANES), and the extended X-ray absorption fine structure (EXAFS). The XANES region offers knowledge about the oxidation state and coordination environment of the element of interest, while the EXAFS region provides structural information about the nature, distance, and coordination number of neighboring atoms. Consequently, these techniques have been extensively employed for the direct analysis of solid samples, including organisms.^214,215^ XAS offers distinct capabilities compared to other As speciation methods, since it facilitates *in situ* As speciation in different sample matrices. This includes crude extracts, frozen hydrated samples freeze-dried samples, and subcellular compartments, regardless of their physical state (solid, liquid, or gaseous). This phenomenon is not accomplished with conventional techniques. In addition, LOD is approximately about 1–10 μg g^−1^ based on the experimental status.^216^

XAS is adaptable with LC-ICP-MS, allowing for the coupling of structural elucidation of innovative compounds such as As-sugars and arsenolipids in their original state in the seafoods. This is particularly valuable in cases where documented structures are postulated from those of known fatty acids or hydrocarbons due to the absence of identification methods and standards.^59,217^ XAS has some drawbacks such as the utilization of hard X-ray beams with high energy, leading to a potential risk of sample damage. Additionally, certain As compounds may have closely situated or identical absorption edges, necessitating the obligatory use of standards. Nevertheless, many standards of organoarsenicals of interest, like As-sugars and arsenolipids are difficult to discover. Metals attached to lighter elements such as O, P, N, and S functional groups are less detectable by XAS. Similar nearest-neighbor conditions for arsenic compounds have comparable white line energies and could be mistakenly recognized in XAS if LC-ICP-MS comparison isn't made. For instance, the white line energy of tetramethylarsonium ion (TETRA), AsB (AsB), and arsenocholine (AsC) is the same which is 11 872.6 eV.^216^

## 10 Quality control

Numerous details regarding this element's bioavailability, toxicity, and the environmental processes can be obtained from the As speciation analysis. However, it is clear that this information is dependent upon precise analytical data being obtained. As a result, during the whole analytical process—from sample to detection—any potential source of mistake, including contamination, species loss or transformation, and identification/quantification errors, must be carefully taken into account.^220^

There are certain quality control standards to adhere to in order to prevent or reduce the effects of species modifications and to ensure the accuracy and dependability of speciation data. Evaluations of mass balance, extraction efficiency, and column recovery in particular are crucial. The mass balance data can indicate whether and where losses in the speciation process take place, as well as information about the distribution of the elements during each analytical phase (extraction, separation, and species detection).^221^ In the last several years, there has been a demand in the need for different certified reference materials (CRM) in chemical analysis, along with new publications about CRM advancements and certification. Because CRMs are used in so many processes, such as method validation, proficiency testing, uncertainty estimation, and quality control, they are emphasized as one of the numerous technological criteria in this quality system.^222^

It is vital to highlight the usage of CRMs as the foremost and potent tool for quality control. Over the past decades, enormous CRMs have emerged in the analytical realm, aiming to encompass various matrices and empower researchers to assess the accuracy of their analytical methods and resulting data. It is recommended that environmental control laboratories utilize CRMs in order to evaluate or verify the precision and accuracy of their analytical techniques, guaranteeing the delivery of accurate data and compliance with legally mandated data quality requirements. Although As-containing CRMs have been created, the majority of them attest to the total-element concentration. Producing CRM materials specific to various As species has become imperative due to the growing demand for species-specific data.^7,223^ In the As speciation analysis of environmental samples, a variety of reference materials were employed, spanning various types of marine and terrestrial organisms, and other biological samples. While the majority of the reviewed papers reported using CRMs for quality control, just few of these studies applied for the validation of arsenic speciation analysis. It is noteworthy that most CRMs are certified for total As only, and certified values for individual species were available for only a few, specifically BCR-627 (AsB, DMA), DORM-2 (AsB, TETRA), and NMIJ 7402 (AsB). Some of used certified materials for As speciation in marine samples are listed in Table 3.

**Table 3:** Some of certified reference materials used to check validity of measuring total As and As species in marine samples

Name of certified reference material	Extraction media	Certified value μg g^− 1^	Obtained value μg g^− 1^	Extraction efficiency (%)	Certified value for As species	Obtained value for As species μg g^− 1^	Extraction efficiency for As species (%)	Reference
DORM-3	10 mmol L^−1^ (NH_4_)_2_HPO_4_	6.88 ± 0.30	6.78 ± 0.21	99				140
BCR 279 sea lettuce (Ulva lactuca)	Water, shaking for 16 h	3.09 ± 0.2	2.9 ± 0.3	94				224
DORM-3-fish protein	Trypsin enzyme in 0.1 mol L^−1^ NH_4_HCO_3_	6.88 ± 0.3	6.94 ± 0.36	101				87
BCR-627	Methanol/water (1 : 1 v/v)				3.90^a^ ± 0.22	4.27^a^ ± 0.23	109	135
Tuna fish tissue BCR-627	Water	4.71 ± 0.72	4.8 ± 0.3	98	52^a^ ± 3	50.77^a^ ± 1.36	98	25
					2.07^b^ ± 0.37	2.0^b^ ± 0.3	103	
Herring tissue MODAS-3	Water	8.52 ± 0.32	9.26 ± 0.81	92				25
CRM 7405-a	Hot water dichloromethane: methanol				10.100^c^ ± 0.500	9.143^c^ ± 0.108	91	120
DORM-4	Hot water dichloromethane: methanol				3.950 a ± 0.360	3.740 a ± 0.326	95	120
TORT-3	Hot water dichloromethane: methanol				54.50^a^ ± 2.500	50.80 a ± 1.254	93	120
TORT-2	HNO_3_	21.6 ± 1.80	19.82 ± 1.10	92				210
DOLT-4	HNO_3_	9.66 ± 0.62	8.76 ± 0.33	91				210

a Certified value of AsB.

b DMA.

c As^V^.

## 11 Conclusions

Studies on marine organisms including fish, shellfish, seaweed and macroalgae are of very interesting and important due to growing consumption of these food chains worldwide. Consumption of these foods is highly recommended to humans because of their high nutritional values supplying essential micro and macro nutrients such as vitamins, minerals and proteins. However, presence of heavy metals may cause potential threat to those who consume them on regular basis. This review presented the most important information including health issues, toxicity and occurrence of one of the most widespread heavy metals which is As. It highlighted the steps of analysis of this potential heavy metal in marine ecosystem. The literature expands to provide methods that have been used to extract As in these food samples using different solvents and techniques correlated to perform this process. Different chromatographic, non-chromatographic methods were discussed specifically developed to separate As species based on food mediums. In addition, coupled techniques including ICP-MS, LC-MS, HPLC/ICP-MS which are specified for detecting those As species also exclusively explained. This review concluded that 53 As species have been found in these marine ecosystem. The quality control still remains one of the biggest challenges to maintain the reliability of the outcome of some of As species that have been found in this marine samples. Another challenge which is the requirement for sophisticated and developed instruments that necessary to identify those As species in these marine samples.

## Data availability

The data underlying this article have been included in the article.

## Author contributions

Bashdar Abuzed Sadee: supervision, writing – original draft, conceptualization, validation, methodology. Yaseen Galali: writing – review & editing, investigation: Salih M. S. Zebari: writing – review & editing, visualization, validation.

## Conflicts of interest

There is no conflict of interest to declare
